# A new prognostic model for lung adenocarcinoma according propionate metabolism related genes: a comprehensive bioinformatic study

**DOI:** 10.1007/s12672-025-03540-w

**Published:** 2025-09-26

**Authors:** Chunmei Liu, Liya He, Zexin Peng, Jianmin Luo

**Affiliations:** 1 Department of Radiation Oncology, The Second Hospital of Hebei Medical University, 215 West Heping Road, Shijiazhuang, 050000 Hebei China; 2 Department of Hematology, The Second Hospital of Hebei Medical University, Shijiazhuang, Hebei China; 3 Department of Oncology, Hebei General Hospital, Shijiazhuang, Hebei China; 4 College of Basic Medicine, Hebei University, Baoding, Hebei China

**Keywords:** Lung adenocarcinoma, Propionate metabolism, Risk score, Immune, Prognosis

## Abstract

**Background:**

The prognostic mechanisms of lung adenocarcinoma (LUAD) remain unclear, while the propionate metabolic pathway has been implicated in promoting tumor growth across multiple cancer types. This study aims to elucidate the mechanistic basis by which the propionate pathway influences LUAD progression at the genetic level.

**Methods:**

The TCGA-LUAD cohort was retrieved from The Cancer Genome Atlas (TCGA), and LUAD-related datasets (GSE13213, GSE72079) were obtained from the Gene Expression Omnibus (GEO). Differentially expressed genes (DEGs) between LUAD and normal tissues were first identified, followed by intersection with propionate metabolism-related genes (PMRGs) to derive DE-PMRGs. After partitioning these genes into training and validation sets, a prognostic risk model was constructed via univariate Cox regression and LASSO regression analysis, which was validated in independent cohorts. Patients were stratified into high- and low-risk groups based on the risk model, followed by gene set variation analysis (GSVA), immune microenvironment profiling, and chemosensitivity prediction.

**Results:**

A total of 166 DE-PMRGs were identified by intersecting 4,403 DEGs with 531 PMRGs. A risk model was constructed using five characteristic genes (LDHA, KYNU, SLC2A1, CFTR, MAOB) via univariate Cox and LASSO analyses. GSVA revealed 18 activated pathways in the high-risk group (e.g., heme metabolism, P53 signaling), versus 14 pathways in the low-risk group (e.g., E2F targets, mTORC1 signaling). Significant differences were observed in 14 immune cell types (e.g., eosinophils, neutrophils) and 4 immune checkpoints (PDCD1LG2, CD274, CD27, IDO1) between risk groups. LDHA, KYNU, and SLC2A1 were significantly positively correlated with activated CD4 + T cells, γδ T cells, and memory B cells, while CFTR and MAOB were associated with 9 immune cell types (e.g., activated B cells, eosinophils). Eight chemotherapeutic agents were identified to correlate with risk scores via drug sensitivity analysis.

**Conclusion:**

This study identifies five propionate metabolism-related genes (LDHA, KYNU, SLC2A1, CFTR, MAOB) that may influence LUAD prognosis, providing a scientific foundation for further mechanistic investigations and potential clinical applications. (Liu C, He L, Peng Z, Luo J, A New Prognostic Model for Lung Adenocarcinoma According Propionate Metabolism Related Genes: A Comprehensive Bioinformatic Study, Abstract Book of MEDLIFE2024 & ICBLS2024 (ISBN:979-8-88599-099-8), 2024.)

**Supplementary Information:**

The online version contains supplementary material available at 10.1007/s12672-025-03540-w.

## Introduction

Lung cancer represents the most life-threatening malignant tumor worldwide, ranking first in incidence among male cancers and second among female cancers, with mortality rates paralleling incidence trends. According to the American Cancer Society, there were approximately 228,150 new lung cancer cases and over 142,000 deaths in the United States in 2019 [1]. Lung adenocarcinoma (LUAD) accounts for approximately 40% of all lung cancers, characterized by high metastatic potential and invasiveness [1, 2]. Due to the lack of effective early diagnostic methods, most LUAD patients are diagnosed at advanced stages, resulting in a dismal 5-year survival rate of only 19% [1, 3]. Thus, identifying biomarkers for prognostic prediction and therapeutic guidance in LUAD is of urgent clinical need.

It has been reported that propionic acid upregulates the surface expression of the immunostimulatory NKG2D ligand MICA/B by applying metabolic changes in colon dividing cells, thereby possessing the potential as an immunoactivated anticancer therapy [4]. At the same time a document reports that propionate upregulates ubiquitin protein ligase 2 (HECTD2) in the HECT domain E3, promoting proteasome degradation of histone lysine N-methyltransferase 2 (EHMT2), thereby inhibiting the growth of colon cancer cells [5, 6]. Propionate inhibits the activation of JAK2-STAT3, causes cell cycle arrest, stimulates ROS production, and then leads to p38 activation and breast cancer cell apoptosis [7]. Recent studies have shown that epithelial-mesenchymal transition (EMT) in non-small cell lung cancer (NSCLC) is negatively correlated with short-chain fatty acid (SCFA) propionate. Propionate exerts independent regulation of EMT marker genes in several NSCLC cell lines, increasing the expression of the key epithelial gene E-cadherin while inhibiting the expression of ZEB1. ZEB1 is a major regulator of EMT features and is involved in the early tumorigenesis and metastasis of NSCLC. Additionally, propionate reduces EMT in lung cancer through extensive chromatin changes, including modifications like H3K27ac and H3K4me1/2/3, thereby adjusting the cell phenotype and driving it toward an epithelial state [8, 9]. Studies have also shown that Dioscin inhibits EMT by inactivating the GSK3β signaling pathway, thereby further reducing the proliferation, migration, and invasion abilities of lung adenocarcinoma cells [10]. Dysregulation of propionate metabolism generates pro-invasive characteristics in breast cancer and lung cancer cells, increasing their metastatic potential. This dysregulation occurs through the downregulation of Methylmalonyl-CoA mutase (MCEE), which is mediated by the SP1/EGR1 transcriptional switch driven by ERK2. The transfer signal driven by its promoter level regulates this process. The loss of MCEE leads to the accumulation of methylmalonic acid (MMA), a byproduct of propionate metabolism, both intracellularly and within tumors. This suggests that alterations in propionate metabolism may contribute to tumor progression and invasiveness [11]. Previous studies have constructed prognostic models for lung adenocarcinoma using single genes such as ALDH2 [12] and ARHGEF6 [13]. They have also used pathways like polyamine metabolism and lipid metabolism. However, propionate metabolism is a key pathway. It regulates tumor invasiveness and the immune microenvironment. Systematic research on its related genes in lung adenocarcinoma prognosis remains unexplored.

Therefore, this study focuses on propionate metabolism-related genes for the first time. Based on TCGA and GEO databases, it combines machine learning algorithms. The study identifies propionate metabolism-related genes (PMRGs) associated with LUAD patient survival. These genes provide potential targets for clinical diagnosis, patient prognosis, and treatment. They also lay a theoretical foundation for in-depth understanding of LUAD mechanisms.

## Materials and methods

### Data sources

The TCGA-LUAD cohort (518 LUAD and 59 normal samples) was downloaded from TCGA (https://portal.gdc.cancer.gov), with 513 LUAD samples having complete survival information. External validation datasets (GSE13213, *n* = 117; GSE72079, *n* = 398) were obtained from GEO (https://www.ncbi.nlm.nih.gov/). A total of 531 propionate metabolism-related genes (PMRGs) with correlation scores > 7 were retrieved from GeneCards (http://www.genecards.org).

### Identifying and enrichment analysis of differentially expressed PMRGs (DE-PMRGs)

Firstly, we calculated the differentially expressed genes (DEGs) between LUAD group and the normal group via “DESeq2” R package in the R language (*adj.p* < 0.05 and |log_2_FoldChange| >1) (version 1.26.0) [14]. Then, those DEGs were intersected with PMRGs to obtain DE-PMRGs via Venn tool. Subsequently, enrichment analysis was performed through the “cluster Profiler” R package (R language version 4.0.2) (*adj.p* < 0.05 and count > 2) [15].

### Creation of the risk model

In the TCGA-LUAD dataset, 513 patients’ DE-PMRGs expression data (FPKM) were used to randomly divide them into an internal training set (360 cases) and an internal validation set (153 cases) in a 7:3 ratio. To identify genes significantly associated with patient survival, we performed Univariate Cox regression analysis using the ‘survival’ package (version 3.1–12). In the training set, univariate Cox proportional hazards regression was applied, with the expression levels of differential propionate metabolism-related genes treated as continuous variables to compare against survival time. Genes were selected based on a p-value < 0.05. Based on the genes selected through univariate Cox analysis, the LASSO algorithm was used to select model genes in the training set, with the parameter family set to binomial and type.measure set to class, to perform LASSO logistic regression. Strongly correlated features were selected, and characteristic genes were screened based on the value of lambda.min. The risk scores were calculated based on those genes following the formula:$\:risk\:score=\sum\:_{n=1}^{n}\left(coefi*Xi\right)$. (Note: Coefi represents the multivariate regression coefficient of the ith gene, xi represents the expression value of the ith gene, and n represents the number of model genes). Subsequently, divide patients into high-risk and low-risk groups based on the calculated median risk score, and then plot the Kaplan-Meier (K-M) curves between the two risk groups, followed by drawing the receiver operating characteristic (ROC) curves of the model. Finally, the internal validation set of the TCGA-LUAD dataset and external validation sets (GSE13213 and GSE72079) were used for validation.

### Creation of a nomogram

Independent prognostic factors were identified via univariate and multivariate Cox regression analyses combining risk scores and clinical features. A nomogram for predicting 1-, 3-, and 5-year survival was constructed using the “rms” package (version 6.1-0), with reliability evaluated via calibration curves and ROC analyses.

### Correlation analysis of clinical characteristics

To assess the relevance between clinical characteristics and risk scores, we analyzed the differences of risk score were between the groups divided based on clinical characteristics. The clinical characteristics includes age (≤ 55/>55), gender (female / male), OS status (living/deceased), stage (Ⅰ/Ⅱ/Ⅲ/Ⅳ), pathologic-T stage (T1/T2/T3/T4), TMB (high TMB/ low TMB), pathologic-M stage (M0/M1), pathologic-N stage (N0/N1/N2/N3), smoking (no smoking/smoking), and radiation therapy (No/Yes). To gain a deeper understanding of the impact of different clinical characteristics on risk scores, we performed stratified analyses to assess the heterogeneity of this relationship in different subgroups (e.g., age, sex, pathologic stage, etc.). Moreover, to explore the relevance between the clinical characteristics and survival rate of LUAD patients, K-M curves were plotted.

### Gene set variation analysis (GSVA)

GSVA enrichment analysis can retain key information without specifying explicit differential gene thresholds, and thus find sets of functional genes that are not very different but have a consistent trend of gene differences without screening for differences. To find common functions of genes and associated pathways within a collection of genes, the 50 marker gene sets were gained through the MSigDB as reference gene sets. The GSVA scores for all enriched pathways in the two risk groups were calculated via “GSVA” R package (version 3.0.3) [16]. Finally, the “limma” package in R language (version 3.46.0) was utilized to contrast the differences in the scores of two risk groups [17], using the low-risk group as a reference. t > 1, we consider the pathway to be activated in the high-risk group, and conversely t<-1, we consider the pathway to be activated in the low-risk group.

### Immune analysis

With the Single-sample GSEA (ssGSEA) algorithm, we can obtain the immune cell types, immune functions, and immune pathway activities for each sample, and then group the samples based on immune activity. ssGSEA calculates the rank value of each gene based on the expression profile file, followed by subsequent statistical analysis. The immune-related gene sets used in this analysis include not only immune cell types but also immune-related pathways and functions, and these gene sets are very comprehensive. By using these immune-related gene sets for analysis, we can accurately determine the immune activity of each sample.

Text in R language was used to compare the differences in immune scores between two risk groups. Then the relevance of differential immune cells with each other, and the relevance between characteristic genes and differential immune cells were evaluated via the Spearman (*P* < 0.05). Immune checkpoint expression and TIDE scores were compared to evaluate immunotherapy sensitivity, with SubMap analysis used to assess response to immune checkpoint blockade (ICB) [18].

### Drug sensitivity analysis

To assessing the sensitivity of conventional chemotherapeutic agents in the two risk groups, the IC50 of chemotherapy drugs was calculated high and low risk groups via the “pRRophetic” R package (version 0.5) [18]. The relevance between the risk score and IC50 of conventional chemotherapeutic agents was calculated via the Spearman algorithm with |Spearman correlation| >0.3 and *p* < 0.05. And the differences of IC50 of screened drugs between the two risk groups were compared by Wilcox rank sum test.

### Statistical analysis

In this study, The R software was used to perform statistical analysis. The Wilcox test used to analyze discrepancy between groups. The *P* < 0.05 was considered to represent a significant difference.

## Results

### Acquisition and enrichment analysis of DE-PMRGs

**Fig. 1:**
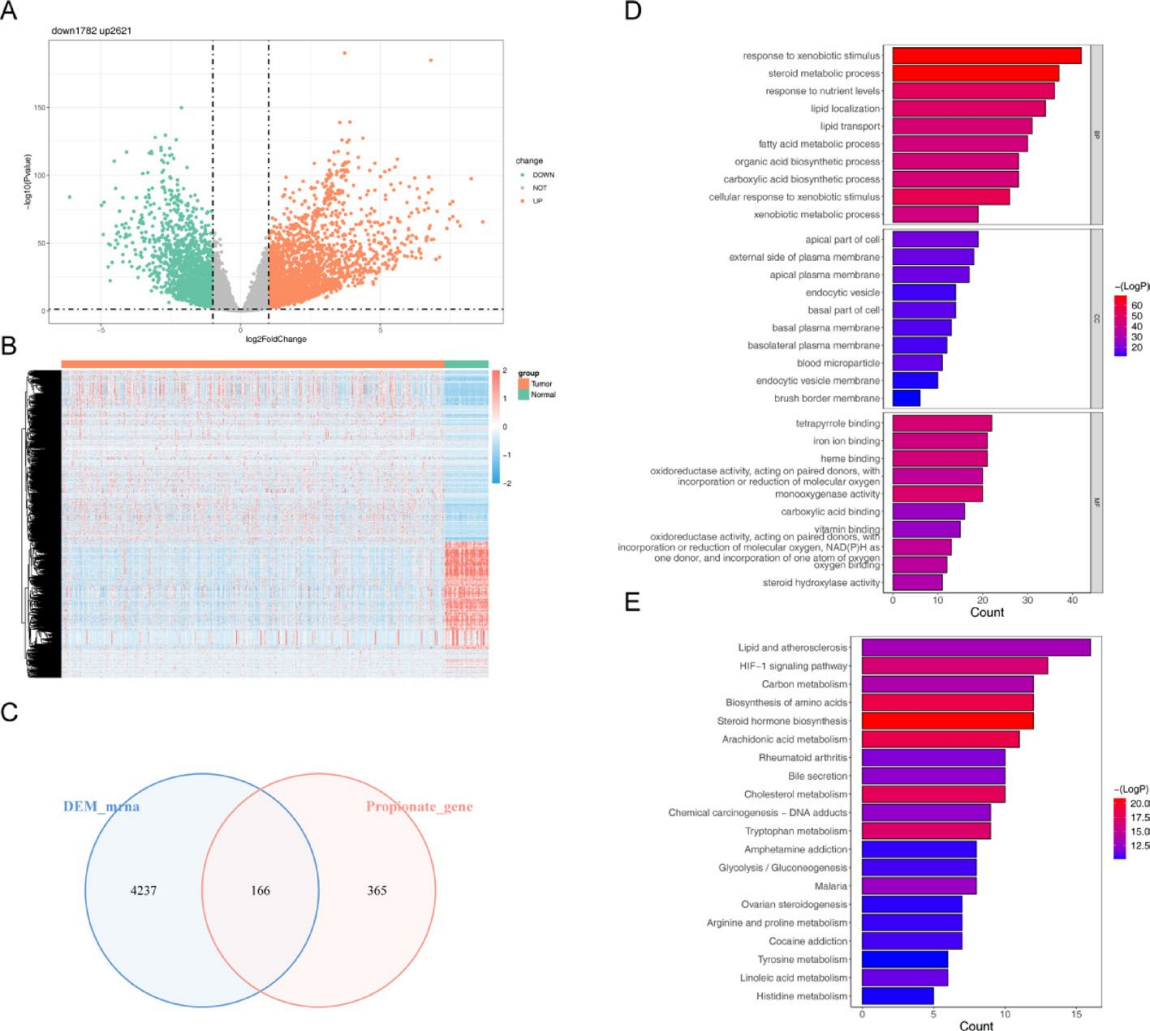
Screening and enrichment analysis of differential propionate metabolism-related genes. (A) Expressing the distribution of differential genes compare the LUAD group with the Normal group using a volcanic map. (B) Expressing the distribution of differential genes compare the LUAD group with the Normal group using a heat map. (C) A Venn chart showing the intersection of DEGs and PMRGs downloaded from the database. (D)GO enrichment map. (E) KEGG enrichment map

A total of 4,403 DEGs were identified between LUAD and normal tissues (Fig. 1A-B), with 166 DE-PMRGs obtained via intersection (Table S1, Fig. 1C). There were 1132 GO items that were enriched (Table S2). For instance, the DE-PMRGs were involved in response to Steroid metabolic process, Apical part of cell, Monooxygenase activity, and so on (Fig. 1D). KEGG analysis identified 62 enriched pathways, such as steroid hormone biosynthesis and arachidonic acid metabolism. (Fig. 1E).

### Establishment and validation of risk model

**Fig. 2:**
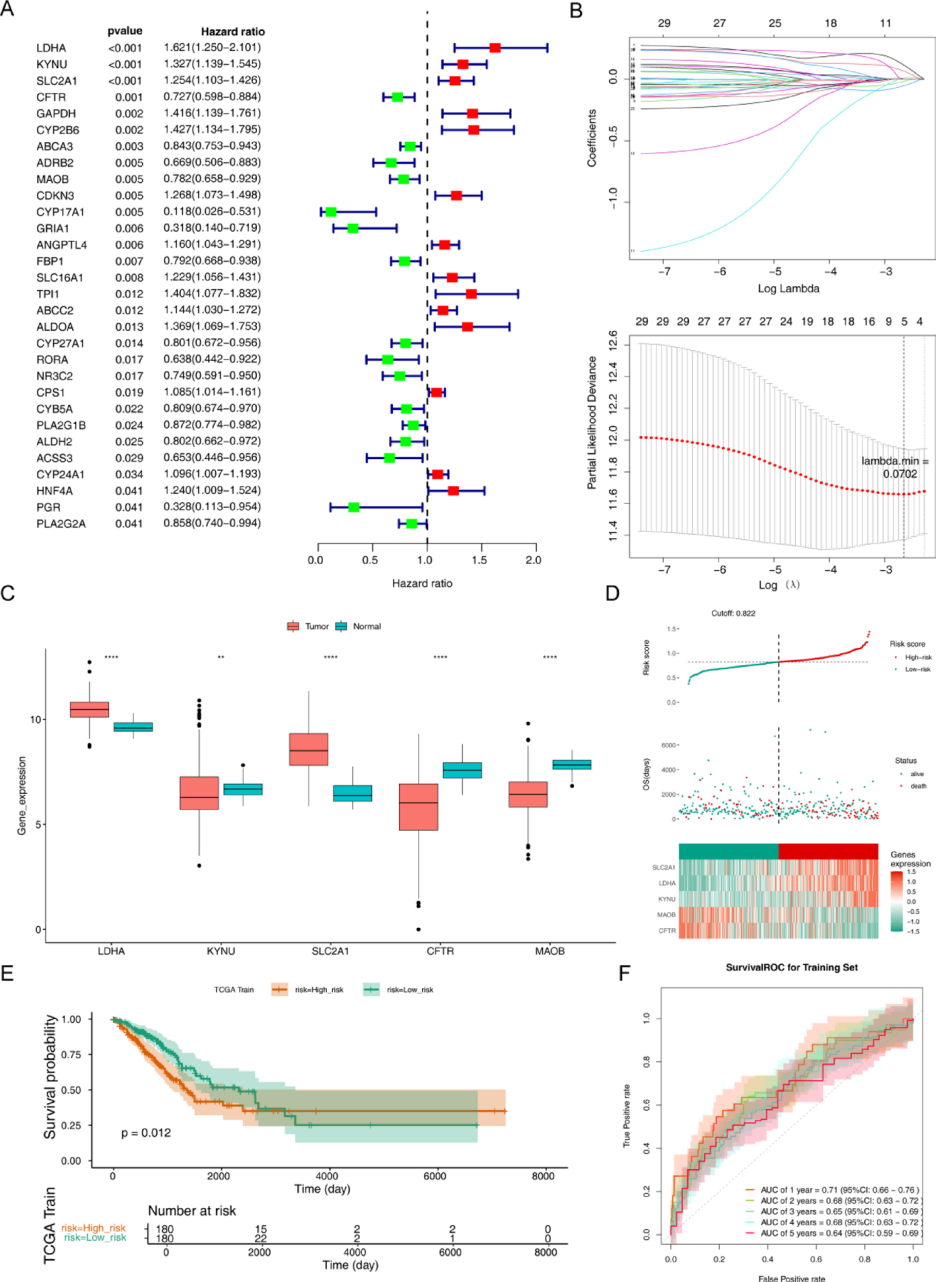
Screening of 5 characteristic genes. (A) Screening prognostic related genes in LUAD patients using univariate Cox regression analysis. (B) LASSO analysis for screening characteristic genes to create risk model. When λ minimum = 0.0702, the mistake of cross-validation is the least, at the same time the characteristic genes are screened out. (C) Compare the expression of characteristic genes in the LUAD group with the Normal group. * P-value < 0.05, ** P-value < 0.01, *** P-value < 0.001,**** P-value < 0.0001, (D) Compare the high risk group with the low risk group by Risk curve, scatter plot, and model gene expression heatmap in the training set .(E) Compare the high risk group with the low risk group by survival analysis in training set.(F) The AUC values were 0.72 (1-year), 0.69 (2-year), 0.67 (3-year), 0.66 (4-year), and 0.61 (5-year), demonstrating that the prognostic prediction of LUAD performed better under the risk model

**Fig. 3:**
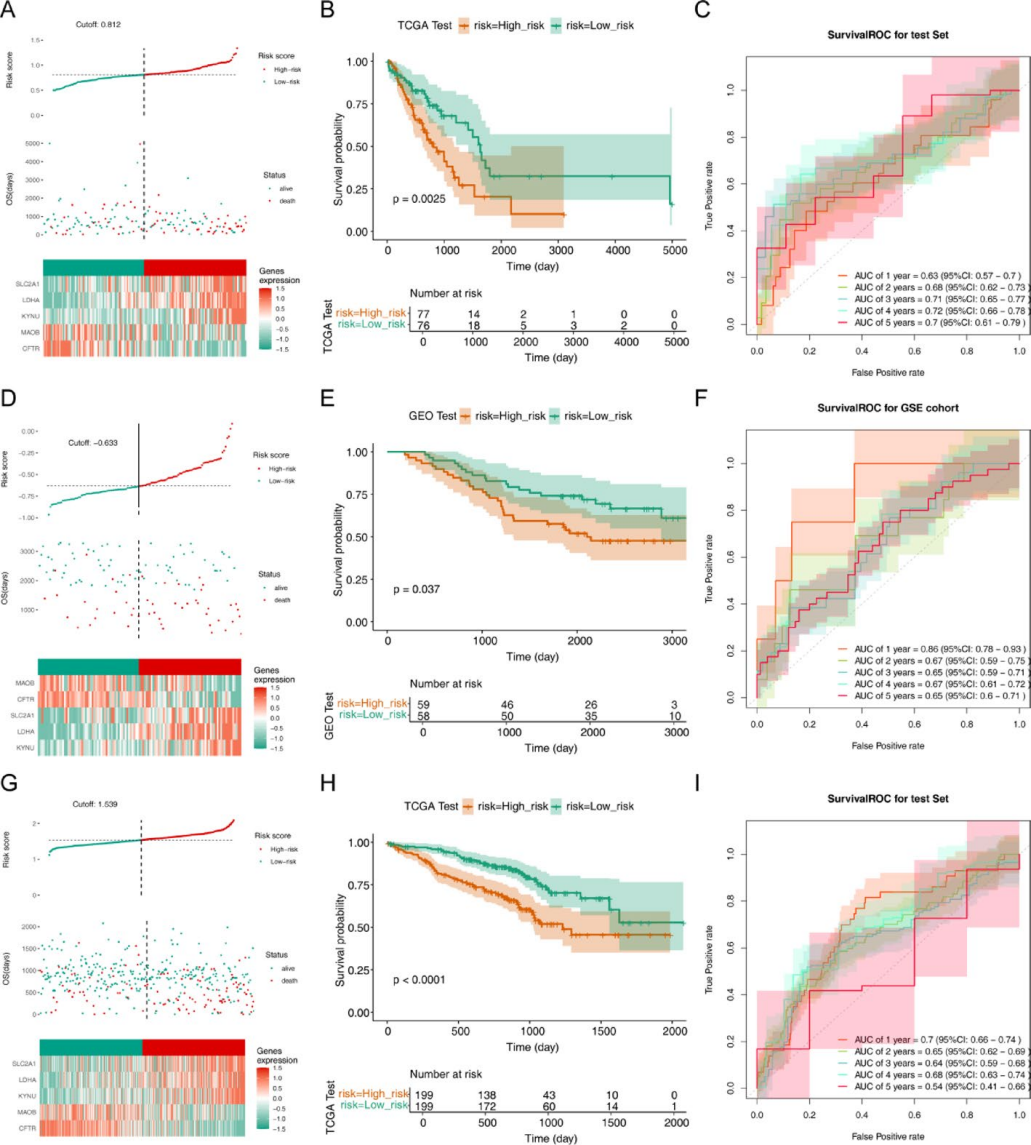
Validation of the risk model based on internal and external validation sets. A, B, C: The internal validation set of TCGA-LUAD dataset. D, E, F: The external validation sets GSE13213. G, H, I: The external validation sets GSE72079. (A) Compare the high risk group with the low risk group by Risk curve, scatter plot, and model gene expression heatmap in the validation set. (B)Survival Analysis between the high- and low-risk groups invalidation set. (C) The AUC values of the gene signature in validation set were showed via the ROC curves. (D) Risk curve, scatter plot, and model gene expression heatmap of the high and low risk groups in the external validation set. (E) Compare the high risk group with the low risk group by survival analysis in external validation set. (F) The AUC values were0.86 (1-year),0.67 (2-year),0.65 (3-year), 0.67 (4-year), and 0.65(5-year) in external validation set, demonstrating that the prognostic prediction of LUAD performed better under the risk model indicating that the risk model had a better performance of the prognostic prediction of LUAD. (G) Compare the high risk group with the low risk group by Risk curve, scatter plot, and model gene expression heatmap in the validation set. (H)Survival Analysis between the high- and low-risk groups invalidation set. (I) The AUC values of the gene signature in validation set were showed via the ROC curves

Through univariate COX’s analysis, we found that a total of 30DE PMRGs were significantly correlated with the prognosis of LUAD patients (*p* < 0.05) (Fig. 2A). There were 5characteristic genes (LDHA, KYNU, SLC2A1, CFTR, and MAOB) that were selected for building a risk model through LASSO analysis (Fig. 2B). RiskScore = − 0.0519697CFTR − 0.01735MAOB + 0.116082LDHA + 0.0669897KYNU + 0.0108580SLC2A1. Moreover, there is a significant difference in the expression of five characteristic genes between the LUAD group and the normal group (Fig. 2C). Subsequently, based on the calculation results of the risk model, we divided patients into high-risk and low-risk groups (Fig. 2D). The survival rate of high-risk group patients is lower than that of low-risk group patients (Fig. 2E). The AUC values were 0.72 (1-year), 0.69 (2-year),0.67 (3-year), 0.66 (4-year), and 0.61 (5-year), suggesting that the risk former had a better function of the prognostic prediction of LUAD. (Fig. 2F). Moreover, we performed a verification process using the internal validation set of TCGA-LUAD dataset and external validation sets GSE13213 and GSE72079. The results showed no significant difference compared to the training set, which demonstrated the broad applicability and reliability of our research findings. (Fig. 3).

### The nomogram was generated by COX analyses

**Fig. 4:**
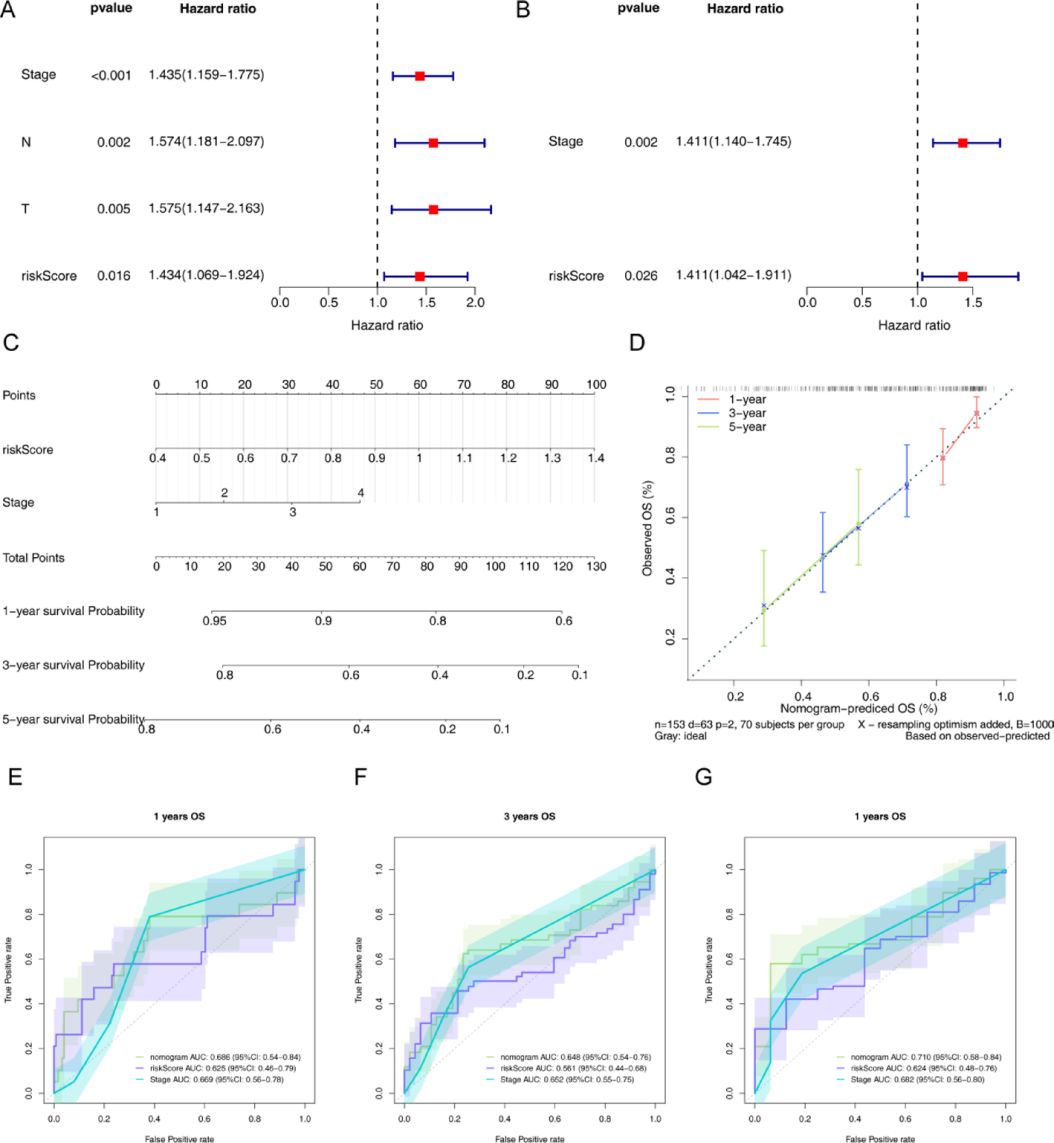
Independent prognostic model constructed based on the training set. (A) The stage, N stage, T stage and risk score were independent prognostic factors for LUAD using Univariate COX analysis. (B) The stage and risk score were independent prognostic factors for LUAD using Multivariate COX analysis. (C) Nomogram contained independent prognostic factors for predicting the OS of in 1year, 3year and5year. (D)Calibration curve of the nomogram (E-G). The AUC values of nomogram were all the highest compare to the risk score and stage in 1year, 3year and 5year

Tumor stage and risk score obtained through calculation formulas were proved that were independent prognosis factors of LUAD via Univariate COX and Multivariate COX analysis (Fig. 4A-B). As shown in Fig. 4C, the nomogram contained the independent prognostic factors. The slope of the calibration curve was 0.7971(1 year), 0.4755 (3 year), 0.3104 (5 year), suggesting that the nomogram had a well forecast effect for LUAD patients (Fig. 4D). The ROC curves of nomogram, tumour stages and different risk groups were plotted separately in 1-year, 3-yearand 5-year (Fig. 4E-G). The results suggested the AUC value of nomogram was all the highest in 1-, 3-, and 5-year, indicating that the nomogram was optimal.

### Correlation between clinical characteristics and risk score

**Fig. 5:**
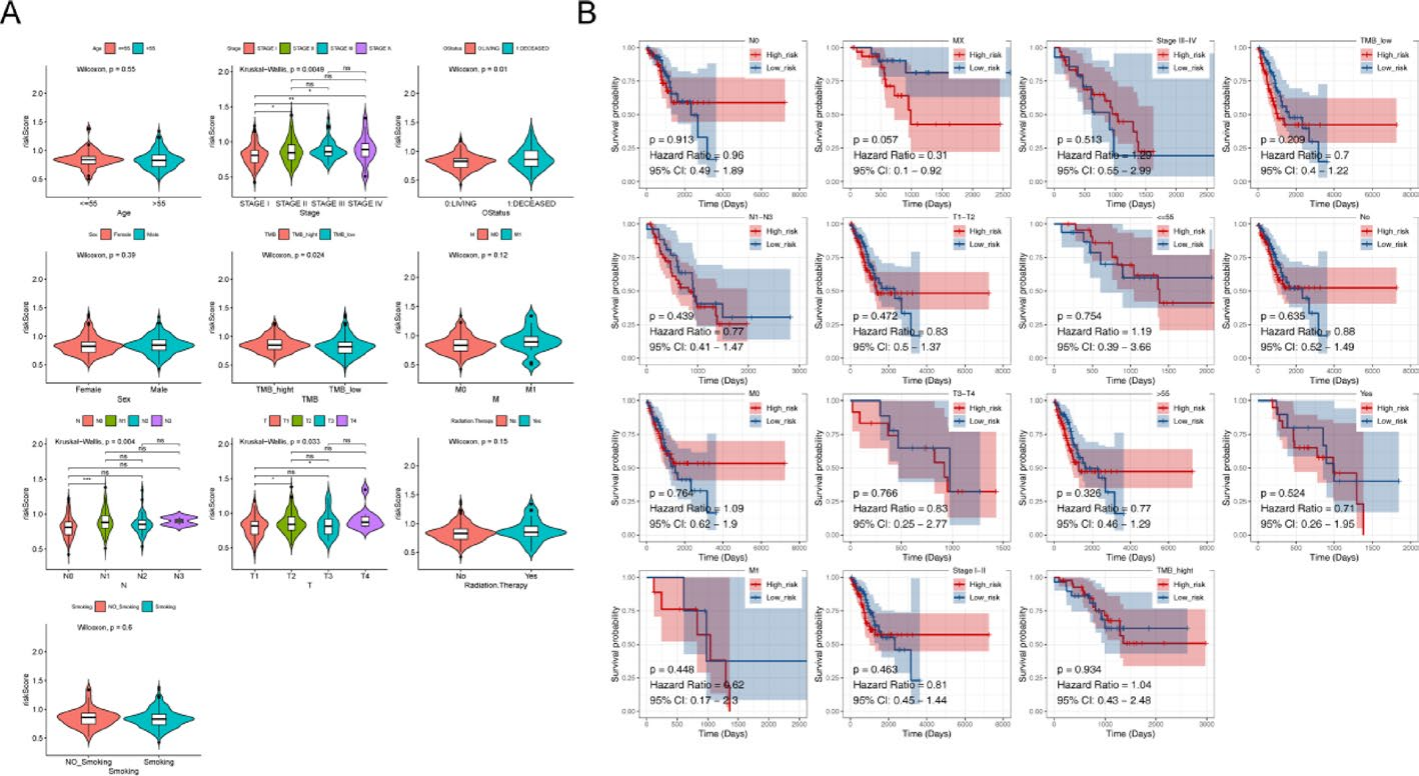
Clinical correlation analysis of high and low-risk groups based on the training set (A) The relativity between risk scores and clinical characteristic s. (B) Stratified survival analysis of clinical grouping and risk models

In order to assess the relevance of the clinical features and risk score, the differences in risk score were compared in the groups divided based on the different clinical characteristics. The results demonstrated that there was marked variability in stage (stage Ⅰ and Ⅱ, stage Ⅰ and Ⅲ, stage Ⅰ and Ⅳ), pathologic-N (N0 and N1), OS status (living and deceased), pathologic-T (T1 and T2, T1 and T4), and TMB (high TMB and low TMB) (Fig. 5A). However, the survival rate was markedly better with age (> 55) and TMB (low-TMB) within 5 years. (Fig. 5B).

### GSVA analysis in the two risk groups

**Fig. 6:**
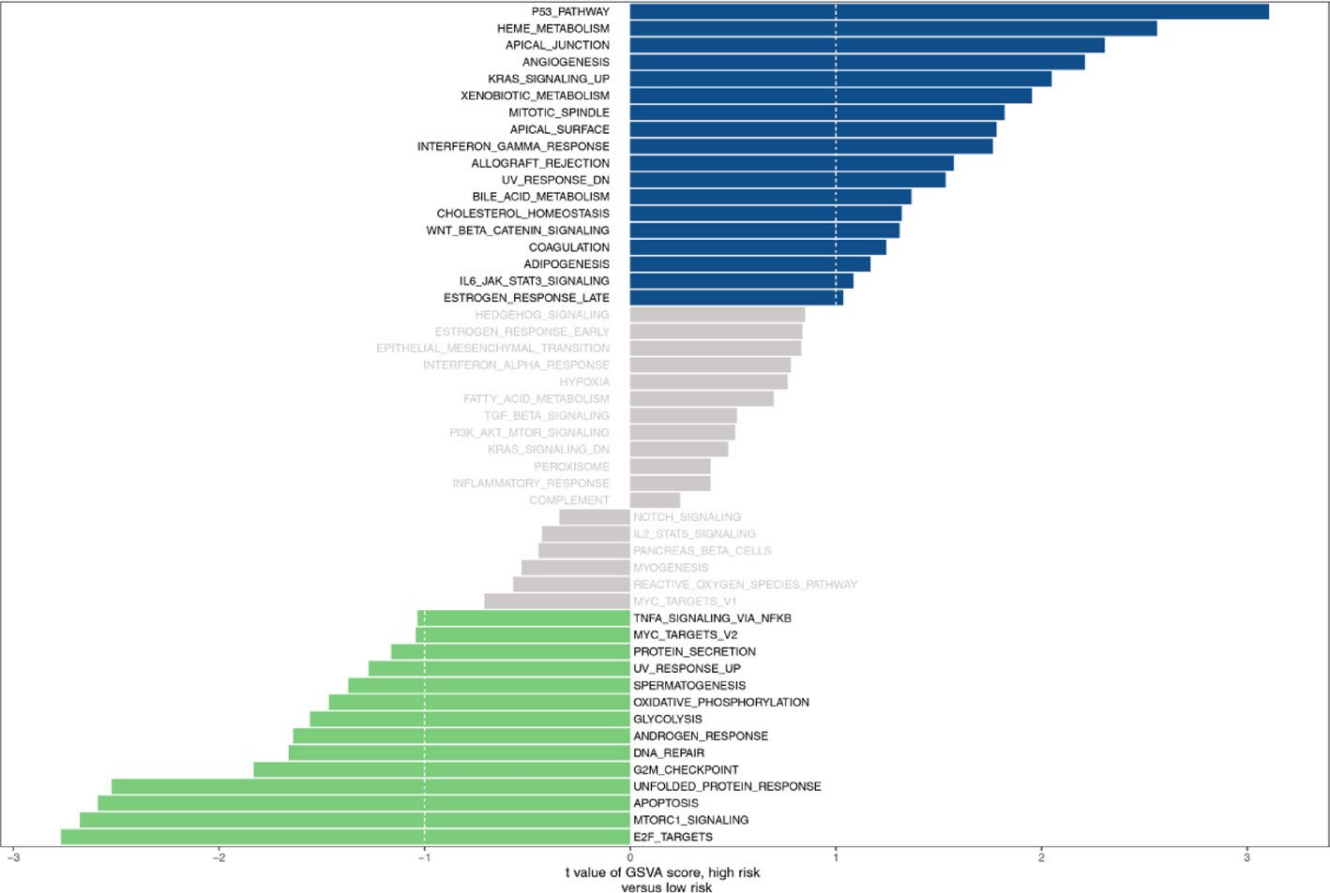
GSVA between high risk groups and low risk groups

In order to analyze the variations in enrichment pathways between two risk groups, we carried out the GSVA. The results demonstrated 18 pathways were enabled in the high-risk groups, for instance, P53 pathways, heme metabolism, apical junction, etc., and 14 pathways were enabled in the low-risk groups, for instance E2F targets, mtorc1 signal, apoptosis, and so on (Fig. 6).

### Immune analysis in the two risk groups

**Fig. 7:**
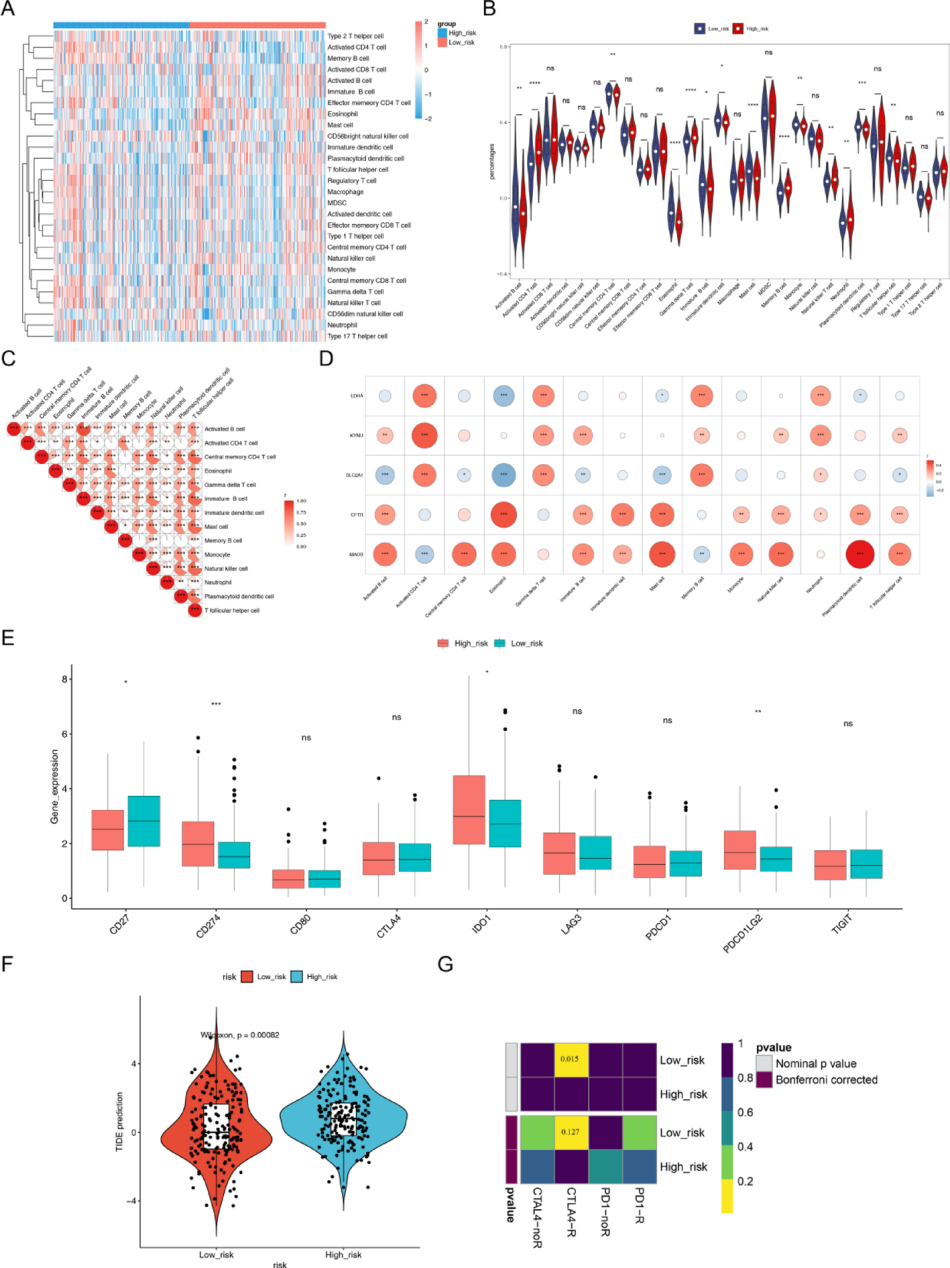
The analysis of immune checkpoint and differential immune cell, which is related to characteristic genes (A) Heat map of immune cell score. (B) Violin plot of functional varies in immune cells comparing the high risk group with the low risk group between high and low risk groups. (C) Correlation results between differential immune cell with each other. (D) Correlation results compare differential immune cells with differential characteristic genes. (E) Expression of the immune checkpoints comparing the high risk group with the low risk group. (F) TIDE scores comparing the high risk group with the low risk group. (G) Evaluation of ICB responses in the immune locus of treatment using SubMap comparing the high risk group with the low risk group. * P-value < 0.05, ** P-value < 0.01, *** P-value < 0.001,**** P-value < 0.0001

The immune level of LUAD samples was explored and demonstrated via heat map (Fig. 7A). There were 14 immune cells with a significantly difference between the two risk groups, such as activated B cell, memory B cell, eosinophil, etc. (Fig. 7B). The correlation results between differential immune cells with each other indicated that the majority of the differential immunocells were positively relevant to each other (Fig. 7C). For instance, immature B cells were highest actively correlated with activated B cell (Fig. 7C). However, only eosinophil was negatively correlated with Memory B cell (Fig. 7C). In addition, LDHA, KYNU, and SLC2A1 were significantly positively related to activated CD4T cell, memory B cell, neutrophil, and γδT cell (Fig. 7D). The CFTR and MAOB were markedly actively relevant to 9 immune cells, for example activated B cell, eosinophil, mast cell, etc. (Fig. 7D). The expression of 4 immune checkpoints (CD27, CD274, IDO1, PDCD1LG2), was markedly differential between the two hazard groups (Fig. 7E). And the TIDE score of a high level was in the high-risk groups (Fig. 7F). And when the immune locus of treatment was CTLA-4, the patients of the low risk level group could achieve higher treatment results (Fig. 7G).

### Drug sensitivity analysis

**Fig. 8:**
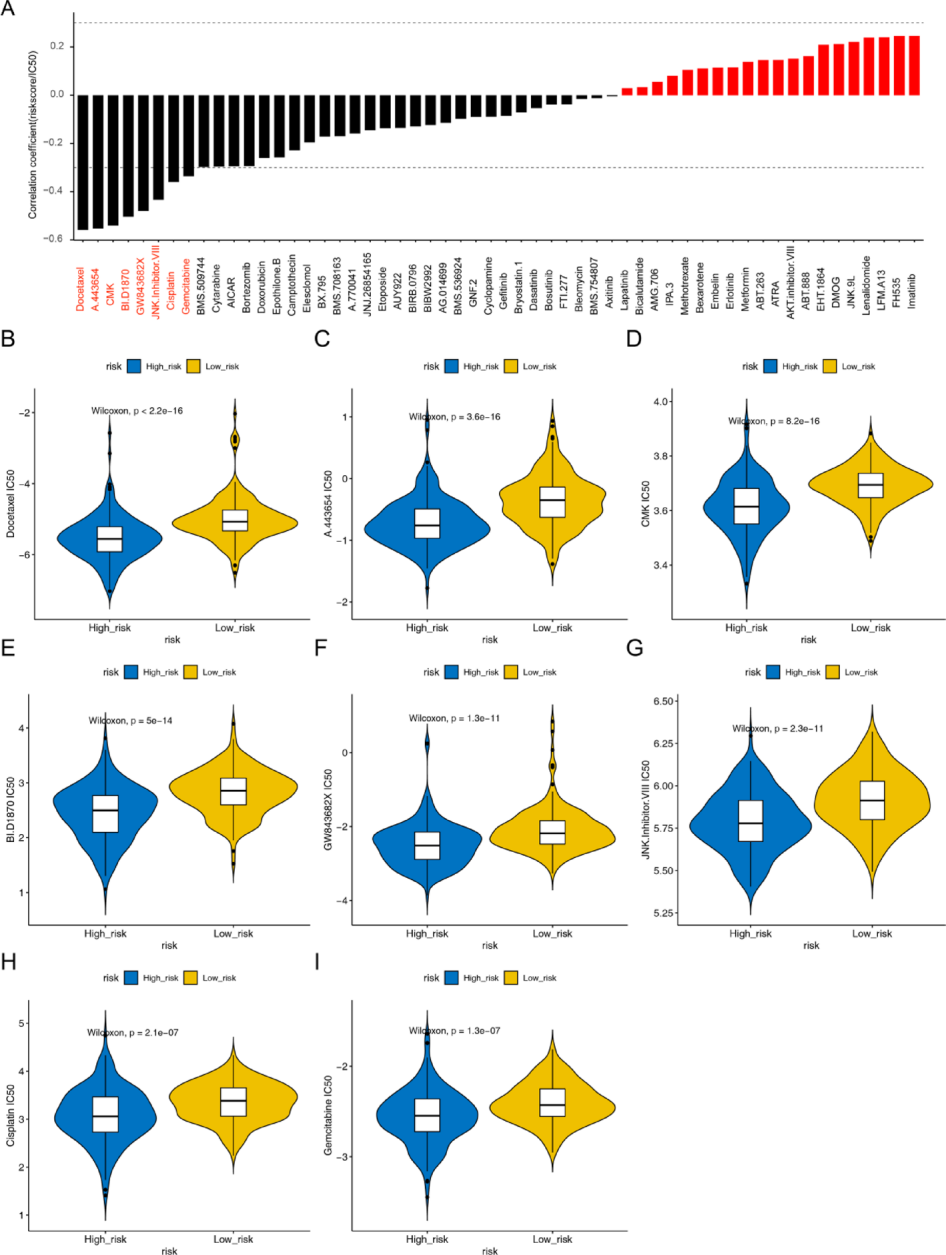
Differential analysis of drug sensitivity and correlation analysis between drug sensitivity and risk score. (A) the IC50 of 8 drugs was negatively relevant to risk sores. The more the Y-axis value, the more the absolute value of the correlation coefficient; The drugs in red font are the key drugs we have screened. (B-I) Analysis of sensitivity differences among eight drugs: Docetaxel, A.443,654, CMK, BI.D1870, GW843682X, JNK.Inhibitor.VIII, Cisplatin, and Gemcitabine. *P* < 0.001

The 8 drugs were screened, namely docetaxel, A.443,654, CMK, BI.D1870, GW843682X, JNK.Inhibitor.Ⅷ, cisplatin, and gemcitabine, and the IC50 of 8 drugs were negatively relevant to risk sores (Fig. 8A). Figure 8B-I illustrated that the differences in treatment efficacy between the high-risk and low-risk groups of 8 commonly used drugs. From these 8 figures, it can be seen that the IC50 of the 8 drugs were higher in the low-risk level groups.

### Bootstrap analysis

**Fig. 9:**
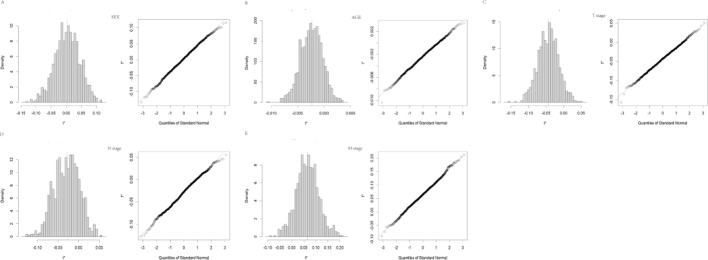
The confidence interval of gender, age, T stage, N stage and M sage. (A) The confidence intervals for gender are (-0.0829,0.0789). (B) age are (-0.0067,0.0016). (C) T stage are (-0.1049,0.143). (D) N stage are (-0.0863,0.0276). (E) M stage are (-0.0324,0.1667)

The BCa method using the boot function of R language was applied to analyze the TCGA-LUAD cohort studied in this paper. It was found that the gender confidence interval of the sample was (-0.0829,0.0789) (Fig. 9A), the age confidence interval was (-0.0067,0.0016) (Fig. 9B), the T staging confidence interval was (-0.1049,0.143) (Fig. 9C), the N staging confidence interval was (-0.0863,0.0276) (Fig. 9D), and the M staging confidence interval was (-0.0324,0.1667) (Fig. 9E). All confidence intervals crossed 0. Considering that the TCGA-LUAD cohort analyzed in this paper has no significant difference from the overall sample, The analyzed data has wide applicability.

## Discussion

The high mortality rate of LUAD underscores the need for improved prognostic tools and therapeutic targets. While previous studies have explored single genes (e.g., ALDH2 [19], ARHGEF6 [13], EFNA2 [20], CFP1 [21], Keap1 [22], ERBB2 [23], EML4 [24], PARP4 [25]) and metabolic pathways (e.g., polyamine metabolism [26], lipid metabolism [27]), this study is the first to investigate propionate metabolism-related genes in LUAD. Zhao found that propionate concentration in lung cancer patients is significantly higher than that in healthy individuals. Propionate can participate in the tricarboxylic acid cycle (TCA) via mitochondria, inhibiting glycolytic reprogramming in tumor cells. As a metabolite of gut microbiota, propionate can enhance the anti-tumor immune response. The study notes that short-chain fatty acids (e.g., propionate) can synergistically improve the efficacy of chemotherapy or immunotherapy (such as anti-PD-1 antibodies). This is achieved by increasing tumor sensitivity or enhancing immune cell function [28]. Iqbal found that propionate reduces intestinal permeability, decreasing lipopolysaccharide (LPS) translocation. This suggests it regulates the lung microenvironment via the “gut-lung axis”, e.g., by reducing pro-inflammatory cytokines (TNF-α, IL-6) and lowering lung cancer risk. Inflammatory microenvironments drive lung cancer development, and propionate’s anti-inflammatory properties may mitigate lung inflammatory damage. This reduces risks of gene mutations and cellular malignant transformation. Propionate promotes regulatory T cell (Treg) differentiation and cytokine secretion (e.g., IL-10), enhancing immune surveillance. Additionally, it improves intestinal barrier integrity, potentially modulating systemic immune cell activity. This boosts natural killer (NK cell) cytotoxicity against lung cancer cells. Propionate negatively correlates with EMT, inhibiting tumor cell invasion and metastasis [29]. Liu’s study analyzed the gut metagenome and metabolome of lung cancer patients initiating anti-PD-1/PD-L1 therapy. It found higher propionate levels (*P* = 0.01) in gut microbiota of the long-term benefit group. Propionate inhibits tumor cell glycolytic reprogramming by activating the AMPK pathway. It promotes fatty acid oxidation, reduces lipid accumulation, and suppresses lung cancer cell proliferation. By activating G protein-coupled receptors (e.g., GPR41/GPR43), propionate enhances CD8 + T cell cytotoxicity. It also promotes regulatory T cells (Treg) to secrete IL-10, optimizing anti-tumor immune responses. The study proposes propionate concentration as a predictor for long-term immune checkpoint inhibitor (ICI) efficacy. Its levels correlate positively with patient progression-free survival (PFS). Fecal microbiota transplantation (FMT) with high-propionate-producing strains (e.g., Akkermansia muciniphila, Bifidobacterium) may enhance ICI efficacy via the propionate pathway [30]. Qian’s study found that QFM (Qingfei mixture) combined with sodium propionate reduced tumor volume by 42% in tumor-bearing mice. This significantly enhanced therapeutic efficacy compared to single-drug groups. High propionate levels were positively correlated with prolonged progression-free survival (PFS) in patients (HR = 2.93, *P* = 0.01) [31]. Kim found that propionate inhibits the cAMP-AMPK-TAK1-NF-κB signaling axis by activating FFAR2. This antagonizes TLR2/3-induced progression of lung adenocarcinoma cells. The findings provide a new strategy for lung adenocarcinoma treatment: FFAR2 agonists (such as propionate) may serve as potential adjuvants to enhance the efficacy of immunotherapy or targeted therapy. This is particularly applicable to lung cancer patients with high TLR2/3 expression [32]. Huang’s study found that propionate may alleviate systemic inflammatory responses. It does so by inhibiting pro-inflammatory signaling pathways (such as NF-κB) and regulating immune cell functions. The study proposes that modulating the airway microbiota to enhance propionate production could be a new strategy. For instance, supplementing “Veillonella” can increase propionate levels. This, in turn, inhibits NF-κB-mediated inflammatory cascade reactions [11]. Botticelli’s study found that fecal propionate levels in NSCLC patients receiving nivolumab were significantly higher in long-term responders (LRs, progression-free survival > 12 months) than in early progressors (EPs). It proposed that propionate levels could serve as a predictive marker for response in NSCLC patients treated with ICIs (immune checkpoint inhibitors). Modulating the gut microbiota to increase propionate production—such as supplementing propionate-producing bacteria or adopting a high-fiber diet—may serve as an adjuvant to enhance ICI efficacy [33]. Therefore, we attempted to conduct genetic analysis through the propionate pathway to predict the prognosis of lung adenocarcinoma. Reveal the core logic of propionate in regulating tumor immunity from the aspects of molecular mechanisms, biomarker screening, and therapeutic target discovery. In our study, we found the characteristic genes that affected lung adenocarcinoma through the propionate pathway included LDHA, KYNU, SLC2A1, CFTR, and MAOB. LDHA, a key enzyme in aerobic glycolysis, catalyzes the conversion of pyruvate to lactate. It has been confirmed to be abnormally overexpressed in gastric cancer, colorectal cancer, breast cancer, thyroid cancer, cervical cancer, and lung cancer [36–49]. Wang’s research found that LDHA is significantly elevated in cisplatin-resistant LUAD cell lines. Inhibiting LDHA expression can significantly enhance cisplatin sensitivity [34]. Wu’s study found that LDHA plays a key role in LUAD progression by driving lactate metabolism and the formation of an immunosuppressive microenvironment. It was proposed that targeting LDHA can enhance antitumor effects through a dual mechanism of “metabolic reprogramming + immune activation”, providing a new target for combination therapy of LUAD. This study shows that LDHA is a risk factor for lung adenocarcinoma, which is consistent with literature reports [35]. KYNU (kynureninase) is a key enzyme in the tryptophan metabolic pathway, involved in the decomposition of kynurenine. Fahrmann’s study found that activation of the NRF2 pathway can upregulate KYNU expression. Highly expressed KYNU alters the metabolic microenvironment of tumor cells, promoting the induction and infiltration of regulatory T cells (Tregs) and increasing the expression of immune checkpoint molecules PD1 and PD-L1 [36]. Cai’s study found that high KYNU expression was significantly associated with shorter overall survival in patients. It was observed that immune infiltration was reduced in tumors with high KYNU expression, with downregulation of CD8 + T cell and dendritic cell markers, as well as decreased activity of the MHC class II antigen presentation pathway. The study proposed that KYNU serves as an immunometabolic target with both prognostic and therapeutic values in LUAD [37]. This is consistent with the result of KYNU as a risk gene in this study. SLC2A1 may indirectly affect the propionic acid pathway by influencing intracellular glucose levels. Sarang et al. showed through survival analysis that patients with high SLC2A1 expression had shorter overall survival, and it is an independent risk factor affecting the prognosis of LUAD patients. The expression level of SLC2A1 is also associated with tumor staging, and the later the tumor stage, the higher the expression of SLC2A1 [38, 39]. Low expression of CFTR is associated with poor prognosis in patients with colon cancer, breast cancer, head and neck cancer, and lung cancer [40]. Pagliaro’s review found that the protein encoded by the CFTR gene is crucial for maintaining normal cellular physiological functions. Its mutations cause abnormal protein function, leading to chronic inflammation. Immune cells like neutrophils infiltrate the airways and release abundant inflammatory factors, such as IL-8, IL-6, and TNF-α. These factors not only cause lung tissue damage but also activate related signaling pathways, promoting tumor cell proliferation, survival, invasion, and epithelial-mesenchymal transition (EMT). Sustained inflammation also increases oxidative stress, generating large amounts of reactive oxygen species (ROS). ROS damage intracellular lipids, proteins, and DNA, causing DNA mutations, activating oncogenes, and driving cellular carcinogenesis, which provides favorable conditions for LUAD development. It was proposed that CFTR modulators may indirectly affect the occurrence and progression of LUAD by regulating inflammatory responses and the composition of the microbiota [41]. Monoamine oxidase (MAO-B) is significantly overexpressed in LUAD tissues. Alsaad’s review shows that MAO-B overactivation increases mitochondrial ROS levels, causing DNA damage, gene mutations (e.g., TP53 mutation), and oncogene activation (e.g., EGFR pathway). Elevated MAO-B activity promotes GLUT1 and HK2 expression, enhancing glucose uptake and lactate production (Warburg effect) to supply energy and metabolic intermediates for cell proliferation. MAO-B-generated ROS inhibits T cell activity and promotes MDSCs and Tregs infiltration, forming an immunosuppressive microenvironment. MAO-B promotes tumor angiogenesis via HIF-1α/VEGF pathway to provide nutritional support for LUAD cells. High MAO-B expression enhances cell resistance to ionizing radiation by activating NF-κB pathway. Selegiline/Rasagiline and Danshensu, by covalently binding to MAO-B active sites, induce cell cycle arrest, apoptosis, and enhance radiotherapy efficacy. Based on MAOB regulatory mechanisms, MAOB inhibitors combined with radiotherapy/immunotherapy are proposed to improve treatment efficacy [42]. In summary, these metabolic genes play significant roles in the occurrence, development, and prognosis of lung adenocarcinoma. Prognostic models based on these metabolic genes can help assess patients’ survival risk more accurately and provide guidance for personalized treatment.

Establish a nomogram prediction model based on these 5 genes and stages, and divide the training group patients into high-risk and low-risk groups according to risk scores. It was found that there was a significant difference in survival rates between the two, and validation was conducted in the validation group, both of which proved the effectiveness of the risk model. Then we conducted a stratified analysis to evaluate the predictive model’s ability in predicting overall survival rates for different subgroups of patients. We found that for patients with TMB-L and over 55 years old, risk scores can predict the overall survival of patients within 5 years, and there is a significant difference between the high and low risk groups. For smoking and T1-2 patients, OS within 5 years can also be well predicted, but it is not significant. For patients with T3-4, N1-3, and M1, it can also be predicted well, but it is not significant. For patients with TMB-H and M0, the OS after 3 years can be well distinguished, but it is not significant. For non-smoking patients, OS within 4 years can be predicted well, but it is not significant. Previous literature has also conducted stratified survival analysis on the clinical predictive ability of the model, and Al Dherasi’s 7-gene model can effectively predict the prognosis of different ages, genders, and stages [43]. Zhao et al. reported that a 15 gene model can effectively predict the prognosis of LUAD patients in stage IA [44]. Wu et al. reported that the 21 gene prediction model can effectively predict LUAD patients in stage IA [45]. It seems that our model has good applicability in predicting the prognosis of different clinical features.

According to GSVA enrichment analysis, P53 pathways, heme metabolism, apical junction, and KRAS signaling pathways, WNT signaling are activated in high-risk groups. These signaling pathways typically involve DNA damage, oxidative stress, activation of proto-oncogenes, vascular growth, and invasion, all of which are related to tumor progression. According to reports, regulating the WNT pathway can reduce the resistance of lung adenocarcinoma cells to osimertinib [46]. Ld1 reduces the resistance of lung adenocarcinoma cells to trametinib by regulating the KRAS pathway [47]. The low risk group activates pathways such as MTORC1 signal, E2F, apoptosis, and MYC targets [48] which are relatively stable and often involve autophagy, apoptosis, and invasion.

The immune microenvironment plays a crucial role in the occurrence, development, and prognosis of lung cancer. Through immune microenvironment analysis, this study found significant differences in immune cell composition and immune checkpoint expression between the high and low-risk groups, including activated CD4 + T cells, central memory CD4 + T cells, eosinophils, etc [49]. Activated CD4 + T cells are crucial for anti-tumor immune responses. By enhancing adaptive immunity against cancer cells, they may improve patient prognosis [50]. Meanwhile, the expression of immune checkpoints such as CD27, CD274 (PD-L1), IDO1, and PDCD1LG2 also showed significant differences between the high and low-risk groups. In particular, the high expression of CD274 (PD-L1) may inhibit T cell function, promote tumor cell immune escape, and lead to poor prognosis in high-risk group patients [51]. Additionally, the upregulation of IDO1 may also suppress T-cell activity, promote tumor immune tolerance, and further exacerbate tumor progression [52]. The two work synergistically to create an immunosuppressive microenvironment, further promoting tumor immune escape [53]. In contrast, the low-risk group exhibits an immune-activated microenvironment with higher levels of activated T cells and lower levels of immunosuppressive molecules, which helps inhibit tumor growth and improve patient prognosis [54]. Notably, previous studies have confirmed that tumor-infiltrating B cells can affect the survival of non-small cell lung cancer patients by regulating the phenotype of CD4 + T cells [55]. These findings indicate that the polarization of the immune microenvironment plays a critical role in the prognosis of lung cancer.

Finally, we also predicted the differences in the therapeutic effects of chemotherapy drugs between the two groups, indicating significant differences in the efficacy of immunotherapy and chemotherapy among different groups, leading to different prognoses.

In summary, the five genes identified all play important roles in the occurrence, development, treatment, and prediction of lung adenocarcinoma (LUAD). By combining these five genes to calculate a risk score and using a Nomogram predictive model, the 1-year/3-year/5-year survival rates of patients can be estimated. Patients with poor prognosis are recommended to initiate combined therapies as early as possible, while those with favorable prognosis can choose milder treatment approaches. This helps clinicians provide personalized treatment recommendations and improves patients’ quality of life. Current clinical prognosis for patients relies mainly on tumor staging and tumor burden, lacking an accurate predictive method. The findings of this study may provide a feasible approach for predicting LUAD prognosis. For patients and families with limited economic conditions, survival rate prediction can help evaluate therapeutic benefits, formulate consumption plans, and assist in deciding whether to continue treatment, avoiding unnecessary family economic burdens.

However, this study has limitations. First, the TCGA and GEO databases used have relatively limited sample sizes, and the lack of external validation with large independent cohorts may affect the robustness and generalizability of the prognostic model. Additionally, the specific molecular mechanisms of propionic acid metabolism in LUAD—especially in tumor growth and immune escape—remain underexplored. Therefore, future research plans include incorporating more clinical datasets to enhance the model’s robustness and generalizability. We also plan to validate related genes’ expression levels through experimental methods (e.g., qRT-PCR, Western blot, animal models, and cell models) and explore their functional roles and underlying mechanisms in LUAD. This will deepen our understanding of the propionic acid metabolic pathway in LUAD and provide more theoretical basis for clinical treatment.

## Conclusion

This study constructed a prognostic model for LUAD based on propionate metabolism-related genes, successfully identifying five characteristic genes (LDHA, KYNU, SLC2A1, CFTR, MAOB) and demonstrating the model’s good performance in predictive ability. The model provides an effective prognostic assessment tool for clinical practice, helping doctors identify high-risk patients to achieve early intervention and closer monitoring. Additionally, the five characteristic genes may offer new potential therapeutic targets for lung adenocarcinoma. Future research can deeply explore the mechanisms of these genes in the occurrence and development of lung adenocarcinoma, as well as their potential as drug targets, opening up new directions for precision medicine.

## Supplementary Information

Supplementary Material 1.

Supplementary Material 2.

Supplementary Material 3.

## Data Availability

Data is provided within the manuscript or supplementary information files. Publicly database was applied in this study: TCGA (https://portal.gdc.cancer.gov/), GEO database (https://ww.ncbinlm.nih.gov/).

## Declarations

### Transparency statement

The lead author Jianmin Luo affirms that this manuscript is an honest, accurate, and transparent account of the study being reported; that no important aspects of the study have been omitted; and that any discrepancies from the study as planned (and, if relevant, registered) have been explained.
